# Notoginsenoside R1 Attenuates Atherosclerotic Lesions in ApoE Deficient Mouse Model

**DOI:** 10.1371/journal.pone.0099849

**Published:** 2014-06-16

**Authors:** Chenglin Jia, Minqi Xiong, Peiwei Wang, Jingang Cui, Xiaoye Du, Qinbo Yang, Wenjian Wang, Yu Chen, Teng Zhang

**Affiliations:** 1 Yueyang Hospital, Shanghai University of Traditional Chinese Medicine, Shanghai, China; 2 Clinical Research Institute of Integrative Medicine, Shanghai University of Traditional Chinese Medicine, Shanghai, China; 3 Institute of Integrative Medicine, Fudan University, Shanghai, China; Max-Delbrück Center for Molecular Medicine (MDC), Germany

## Abstract

**Aims:**

Atherosclerosis is the primary cause of cardiovascular diseases and stroke. The current study evaluated the interventional effects of a naturally occurring compound Notoginsenoside R1 (NR1) on atherosclerosis in ApoE^−/−^ mice.

**Methods and Results:**

The atherosclerotic lesion was significantly alleviated by NR1 treatment and this attenuation was marked by reduction in lipid deposition, fibrosis and oxidative stress. Increased serum levels of GSH and SOD and decreased level of MDH were observed in NR1-treated ApoE^−/−^ mice. NR1 treatment also significantly decreased the levels of CHO, TG, ox-LDL and increased the level of HDL. Additionally, the levels of inflammatory cytokines including IL-2, IL-6, TNF-α and γ-IFN were markedly reduced in NR1-treated ApoE^−/−^ mice. Furthermore, significantly increased aortic expression of miR-26a, miR-21, miR-126a, miR-132, miR-146 and miR-155 and decreased expression of miR-20a and miR-92a were observed in the vehicle-treated ApoE^−/−^ mice. While NR1 treatment led to a significant reduction in the expression of miR-21, miR-26a, miR-126 and increased expression of miR-20a.

**Conclusion:**

Collectively, our results demonstrated for the first time the anti-atherosclerotic effects of NR1, which could be in part mediated through its multiple targeting effects on inflammation, oxidative stress, lipid metabolism and microRNA expression. These results therefore justify further evaluation of NR1 as a therapeutic agent treating atherosclerosis.

## Introduction

Atherosclerosis is a pathological condition of the large arteries characterized by accumulation of lipids and fibrous lesions. The clinical significance of atherosclerosis is heightened by its primary association with the development of cardiovascular and cerebrovascular diseases. Dyslipidemia is of primary importance in the development of atherosclerosis [1]. On the other hand, mainly by removing the excess cholesterol from the peripheral tissue and inhibiting lipoprotein oxidation, HDL is highly protective against atherosclerosis [2]. The level of HDL also relates to the cardiovascular risk in that 10 mg·L (−1) increase in serum concentrations of HDL could decrease cardiovascular risk by 2% to 3% [3], [4]. Atherosclerotic lesions develop from inflammatory and fibroproliferative responses to various insults to the endothelial and smooth muscle cells [1], [5]. Among the specific cellular and molecular responses associated with the pathogenesis of atherosclerosis, fundamental roles of inflammation and oxidative stress has been recognized bridging the risk factor and the mechanisms of atherogenesis. Ongoing inflammatory response, increased production of reactive oxygen species (ROS) and oxidation of low-density lipoprotein (LDL) are noted throughout all stages of atherosclerosis [6]–[8].

microRNAs (miRNAs) are highly conserved noncoding RNAs around 22 nt in length that exert gene expression regulatory functions through post-transcriptional effects. miRNAs bind to the target sequence mostly located on the 3′-untranslated region of the target mRNAs, which leads to translational repression or mRNA degradation. Each miRNA modulates the expression of tens to hundreds of genes, which could result in an efficient and coordinated regulation of multiple cellular pathways, which enables miRNAs to participate in a variety of biological processes, including cellular growth, proliferation, differentiation and the others. Aberrant expression of miRNAs contributes to many pathophysiological conditions including immune and inflammatory responses, aging, cancer, and cardiovascular disease [9]. To be noted, in relation to atherogenesis, miRNAs are connected to inflammatory response, oxidative stress and angiogenesis, etc, processes that are implicated in atherosclerosis. Therefore, miRNAs are gaining increased attention in elucidating the mechanisms leading to atherosclerosis [10]–[12].

Panax notoginseng has been widely used as a medicinal herb for over thousands of years in China. Its clinical application includes treatment of vascular disorders. Anti-inflammatory activity is one of the well-studied mechanisms that contribute to the actions of Panax notoginseng in treating vascular disorders [13]. Panax notoginseng saponins (PNS) are the biologically active constituents accountable for most of the therapeutic effects of Panax notoginseng. Several independent studies have unanimously reported that PNS treatment could alleviate the size of the atherosclerotic lesion in part through improving the blood lipid profile and reducing inflammatory responses [14], [15]. However, which component is responsible for the anti-atherosclerotic action of PNS remains to be clarified. Notoginsenoside R1 (NR1) is the main constituent of Panax notoginseng that possesses cardiovascular activity [16] and unlike other pharmacologically active saponins that are present in both Panax notoginseng and most of other types of ginsengs, NR1 is the unique saponin solely contained by Panax notoginseng. Most important, anti-inflammatory and anti-oxidative activities of NR1 has been noted to be responsible for its therapeutic effects on a variety of disease conditions, including Ischemia-reperfusion (I/R) injury of the kidney [17] and intestine [18] as well as LPS-induced septic shock, etc [19]. However, it is intriguing to find out if this anti-inflammatory activity of NR1 could enable it to be partly responsible for the anti-atherosclerotic effects of PNS. To this end, our current study examined the effects of NR1 on atherosclerosis lesions in ApoE deficient (ApoE^−/−^) mouse model that spontaneously develops atherosclerotic lesions recapitulating the human pathologies [20]. In addition to assessing the interventional effect of NR1 treatment on atherosclerosis and related inflammatory changes and oxidative stress, we further evaluated the effects of NR1 on the expression of miRNAs that are mechanistically associated with atherogenesis.

## Methods

### Animals

Eight-week old male ApoE^−/−^ mice (C57BL/6 genetic background) and C57BL/6 wide type (WT) mice were obtained from Vital River Laboratory Animal Technology Co., Ltd (China). All the mice were housed under a 12 h light/dark cycle and supplied with normal chow ad libitum. At the age of 9 weeks, ApoE^−/−^ mice were switched to a western diet (normal chow supplemented with 20% fat and 1.5% cholesterol) and randomly divided into vehicle-treated ApoE^−/−^ group (n = 9) and NR1-treated groups (n = 9). NR1-treated ApoE^−/−^ mice received daily intraperitoneal injections of NR1 (Batch No. 20110623, Shanghai Source Biological Technology Co., Ltd. China) at the dose of 25 mg/Kg body weight (bw) for eight weeks, while vehicle-treated WT controls and ApoE^−/−^ mice received vehicle in a similar manner. Animals were housed in a standard polypropylene cage containing sterile bedding under a controlled condition of temperature, humidity, and light (12-h light/dark cycle) in the laboratory animal center in Yueyang Hospital, Shanghai University of TCM (No. SYXK [Hu] 2011–0109). All animal handling and experimental procedures were approved by the Shanghai University of TCM Institutional Animal Care and Use Committees.

### Histological examination

At the end of the experiment, after sacrificing the animals, the thoracic aortas and the aortic roots were dissected and fixed by 4% paraformaldehyde for further cryosectioning and paraffin sectioning, respectively. For atherosclerotic lesion examination of thoracic aortas, serial paraffin sections in the thickness of 4 µm were stained with hematoxylin and eosin (H&E). For the examination of the atherosclerotic lesions in the aortic roots, serial frozen sections in the thickness of 12 µm were stained with H&E, oil red O and Masson's trichrome, respectively. For H&E analysis, manual tracing of the entire intima lesion area and area of vessel lumen was performed and the relative lesion area was obtained by calculating the ratio of the lesion area and the area of the vessel lumen. For oil red O and Masson's trichrome staining, the lesion area with positive staining was recorded for analysis. All the analyses were performed by recording the data from 4 equally spaced aortic root sections collected from each animal. Eight mice from each group were utilized for histological examinations. All the sections were examined and recorded by light microscopy (Leica, Germany), which was followed by quantification of the atherosclerosis lesions in the aortic root using Image Pro Plus 6.0 image analysis software.

### Immunohistochemical examination

For immunohistochemistry (IHC), cryosections in the thickness of 10 µm were prepared from OCT-embedded tissue blocks and stained with indicated antibodies. Primary antibodies utilized for IHC examination included α-smooth muscle actin (α-SMA) (Sigma, USA), FITC-conjugated F4/80 (BD, Biosciences, USA). The sections were examined and the photomicrographs were recorded using fluorescent microscope (DM6000B, Leica, Germany).

### In situ production of reactive oxygen species

Dihydroethidium (DHE) (Life Technologies, USA) was administered to the mice via intraperitoneal injection at the dose of 10 mg/Kg bw. All the mice were sacrificed 2 hours after DHE administration and cryosections from aortic root were collected and visualized by fluorescence microscopy (DM6000B, Leica, Germany).

### Measurement of the levels of SOD, GSH, MDH and ox-LDL in serum

Blood samples were collected in EDTA-coated tubes by retro-orbital venous plexus puncture. The serum samples were prepared by centrifugation of blood samples at 1000 g for 10 min. Serum samples were subjected to analyses of the levels of SOD, GSH, MDH and ox-LDL, respectively. The serum concentrations of SOD, GSH and MDH were measured by commercial assay kit following the manufacturer's instructions (Jiancheng Bioengineering Institute, Nanjin, China). The level of serum ox-LDL was determined using ELISA kit according the manufacturer's instructions (Wuhan ColorfulGene Biological Technology. Co., LTD, China).

### Measurement of the serum lipids and inflammatory cytokines

The lipid panel was examined to measure the levels of total cholesterol (CHO), triacylglycerol (TG), low-density lipoprotein (LDL) and high density lipoprotein (HDL). The serum concentrations of IL-2, IL-6, TNF-α and γ-INF were measured by ELISA assays (Wuhan ColorfulGene Biological Technology. Co., LTD, China) according to the manufacturer's instructions.

### Real-time PCR

Total RNA were extracted from thoracic aorta with RecoverAll™ Total Nucleic Acid Isolation Kit (Life technologies, USA) following the manufacturer's protocols. Reverse transcription was then performed using miScript Reverse Transcription Kit (QIAGEN, Germany) following the manufacturer's instructions. Real-time quantitative RT-PCR was done with miScript SYBR Green PCR kit (QIAGEN, Germany). The RCR reactions were programmed as the following: 95°C for 15 min, 40 cycles of 94°C for 15 sec, 55°C for 30 sec and 70°C for 30 sec using LightCycler 480 II (Roche Diagnostics Ltd, Rotkreuz, Switzerland). Primers used for real-time PCR are listed in **Table S1**. Results were analyzed using the comparative Ct method for the relative quantitation of miRNA expression.

### Statistical analysis

The data are presented as means ± S.E.M and statistical comparisons between groups were performed using Student's *t*-test or one-way ANOVA with *p* value less than 0.05 being considered as statistically significant.

## Results

### NR1 attenuated the atherosclerotic lesion in ApoE^−/−^ mice

**Figure 1:**
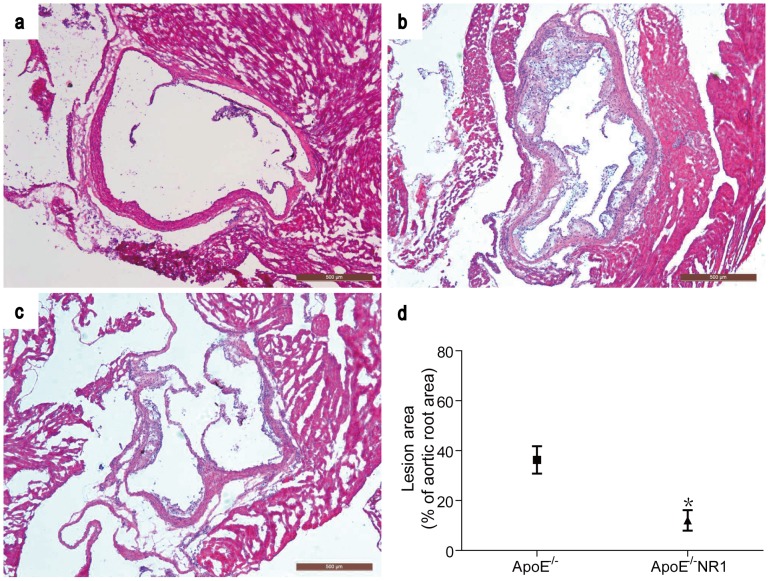
NR1 treatment alleviated the atherosclerotic lesion formation in the aortic root in ApoE^−/−^ mice. Serial cryosections of the aortic roots from vehicle-treated WT control (**a**), vehicle-treated ApoE^−/−^ mice (**b**) and NR1-treated ApoE^−/−^ mice (**c**) were stained with H&E and observed under light microscopy. The size of atherosclerotic lesions in vehicle-treated ApoE^−/−^ mice and NR1-treated ApoE^−/−^ was measured and quantified (**d**). * ApoE^−/−^NR1 vs. ApoE^−/−^, *p*<0.05. Scale bar = 200 µm.

The effect of NR1 on atherosclerosis lesion formation in ApoE^−/−^ mice was first examined by treated the mice with NR1 at the dose of 25 mg/Kg bw for 8 weeks. Histological examination of thoracic aortic roots was performed by H&E staining. As shown in **Figure 1**, atherosclerotic lesion was readily detected in aortic roots in vehicle-treated ApoE^−/−^ mice (**Figure 1a**) compared to the normal vascular histology of aortic roots from vehicle-treated WT controls (**Figure 1b**). In distinct contrast to that from the vehicle-treated ApoE^−/−^ mice, atherosclerotic lesion was noted to be alleviated in NR1-treated ApoE^−/−^ mice (**Figure 1c**), Further quantification of the atherosclerotic lesion in the aortic root showed that the extent of atherosclerosis lesion was significantly reduced in NR1-treated ApoE^−/−^ mice compared to that from the vehicle-treated ApoE^−/−^ mice (12.09±4.10% in NR1-treated mice vs. 36.30±5.47% in vehicle-treated mice, *p* = 0.018) (**Figure1d**). Similar observations were made when serial sections from thoracic aorta were analyzed (**Figure S1**).

### The effect of NR1 on atherosclerotic composition in ApoE^−/−^ mice

**Figure 2:**
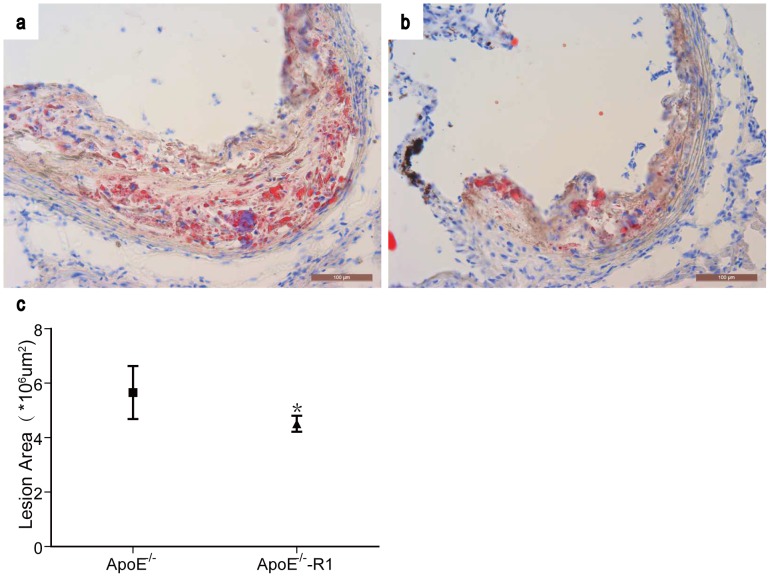
NR1 reduced the lipid deposition in the aortic lesions in ApoE^−/−^ mice. Cryosections of the aorta roots from vehicle-treated ApoE^−/−^ mice (**a**) and NR1-treated ApoE^−/−^ mice (**b**) were examined for the lipid deposition in atherosclerotic lesions by oil red O staining and observed under light microscopy. The oil red O positive staining in the atherosclerotic lesions was measured and quantified (**c**). * ApoE^−/−^NR1 vs. ApoE^−/−^, *p*<0.05. Scale bar = 100 µm.

**Figure 3:**
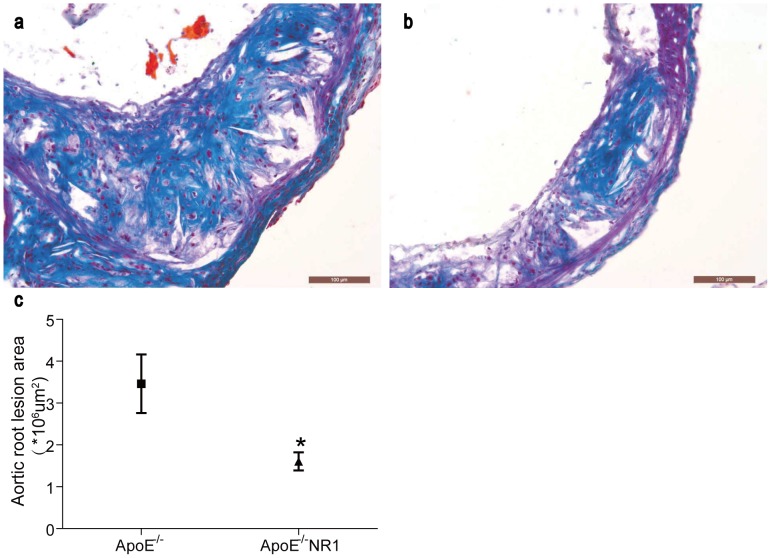
NR1 attenuated fibrosis in the artery intima in ApoE^−/−^ mice. Sections from the vehicle-treated ApoE^−/−^ mice (**a**) and NR1-treated ApoE^−/−^ mice (**b**) were examined for the atherosclerotic fibrosis in the aortic root by Masson's trichrome staining. The Masson's trichrome positive staining in the atherosclerotic lesions was recorded by light microscopy and quantified (**c**). * ApoE^−/−^NR1 vs. ApoE^−/−^, *p*<0.05. Scale bar = 100 µm.

Furthermore, sections of the aortic root were examined by oil red O to evaluate the extent of the lipid deposition in atherosclerotic lesions in ApoE^−/−^ mice with or without NR1 treatment. As shown in **Figure 2**, oil red O positive lipid was detected in the atherosclerotic lesion in both vehicle-treated ApoE^−/−^ mice (**Figure 2a**) and NR1-treated ApoE^−/−^ mice (**Figure 2b**). Quantification of the oil red O positive staining showed that the area of lipid deposition was 5.66±0.21 um^2^×10^6^ per section in vehicle-treated ApoE^−/−^ mice and 4.51±0.29 um^2^×10^6^ per section in NR1-treated ApoE^−/−^ mice (*p*<0.05) (**Figure 2c**), indicating that NR1 treatment led to decreased lipid deposition in the atherosclerotic lesions. The effect of NR1 treatment on fibrotic element of the atherosclerotic lesion was also examined by Masson's trichome staining. The atherosclerotic lesions appeared to be less stained by Masson's trichrome in NR1-treated ApoE^−/−^ mice (**Figure 3b**) compared to that from the vehicle-treated ApoE^−/−^ mice (**Figure 3a**), which was confirmed by quantification of Masson's trichrome positive area in atherosclerotic lesions. As shown in **Figure 3c**, the size of the fibrotic area was 3.72±0.44 um^2^×10^6^ per section in vehicle-treated ApoE^−/−^ mice whereas it was 1.61±0.22 um^2^×10^6^ per section in NR1-treated ApoE^−/−^ mice (*p*<0.05). These data demonstrated that NR1 treatment resulted in significant attenuation of atherosclerosis lesion, reducing the lipid deposition and artery intimal fibrosis in ApoE^−/−^ mice.

### Effects of NR1 treatment on oxidative stress in ApoE^−/−^ mice

**Figure 4:**
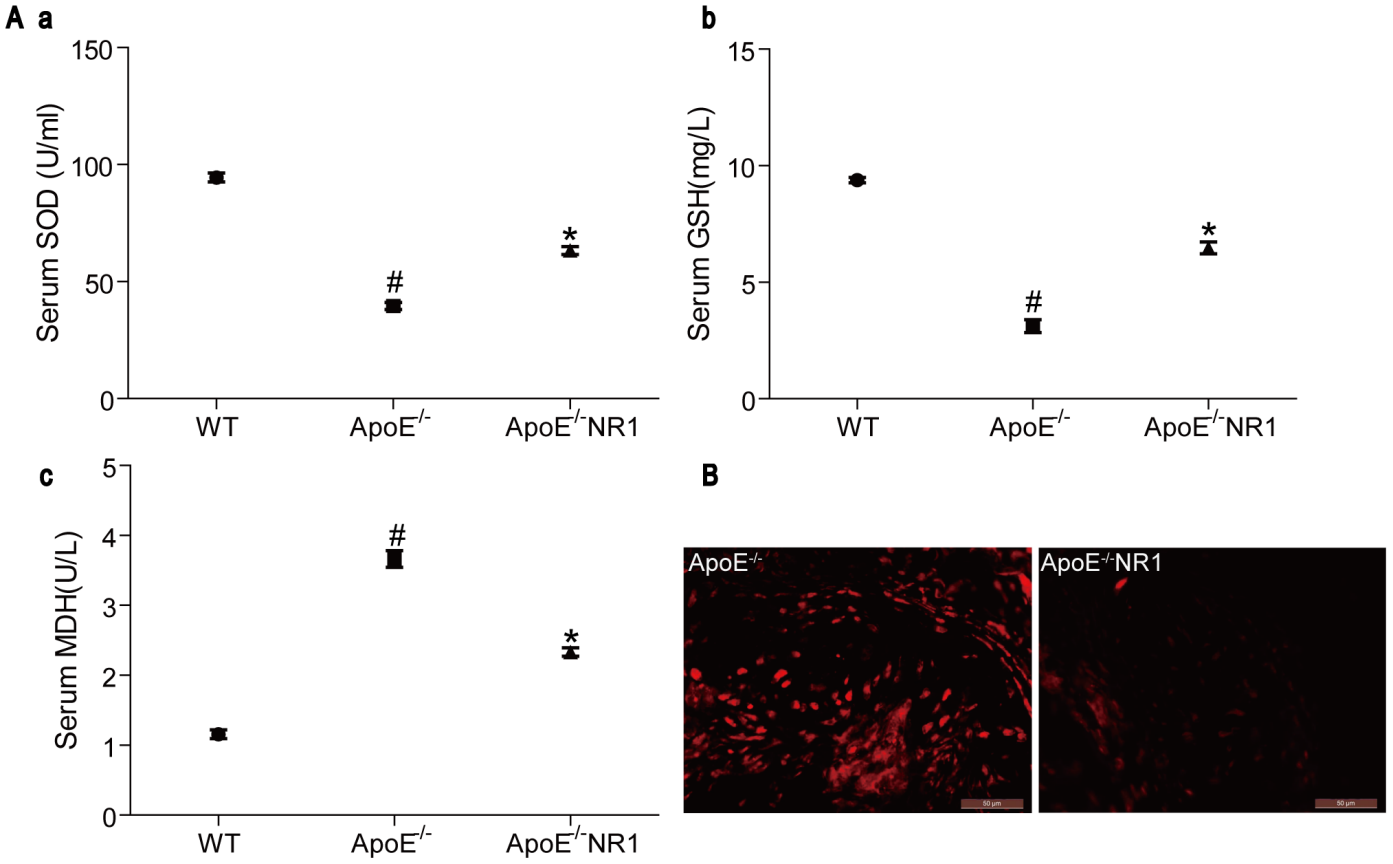
NR1 treatment attenuated oxidative stress in ApoE^−/−^ mice. (**A**) The activities of SOD, GSH and the level of MDH from vehicle-treated WT mice, vehicle-treated ApoE^−/−^ mice and NR1-treated ApoE^−/−^ mice were measured (^#^ ApoE^−/−^ vs. WT, *p*<0.01, * ApoE^−/−^NR1 vs. ApoE^−/−^, *p*<0.01). (**B**) Following the administration of DHE probe, cryosections of the aortic roots from vehicle-treated ApoE^−/−^ mice and NR1-treated ApoE^−/−^ mice (ApoE^−/−^NR1) were examined for the ROS production under fluorescence microscopy. Scale bar = 50 µm.

Next, the effect of NR1 treatment on oxidative stress was evaluated in ApoE^−/−^ mice. The serum levels of SOD, GSH and MDH were measured to directly evaluate the effects of NR1 on oxidative stress in ApoE^−/−^ mice. Compared to that in the vehicle-treated WT controls, the serum levels of anti-oxidative SOD and GSH were significantly reduced in vehicle-treated ApoE^−/−^ mice (*p*<0.01). However, NR1 treatment led to a significant elevation in the serum levels of SOD (**Figure 4A** **.a**) and GSH (**Figure 4A** **.b**) compared to that from the vehicle-treated ApoE^−/−^ mice (*p*<0.01). On the other hand, NR1 treatment decreased the level of MDH in ApoE^−/−^ mice (*p*<0.01), which was significantly increased in vehicle-treated ApoE^−/−^ mcie compared to that from the vehicle-treated WT controls (*p*<0.01) (**Figure 4A.c**). The in situ production of ROS in aortic lesions was also examined by administering the ROS probe, DHE to the mice, followed by fluorescent microscopic analysis of the aortic root sections. As shown in **Figure 4B**, nucleus staining of bright red fluorescence indicative of ROS production was readily detected in the atherosclerotic lesions in vehicle-treated ApoE^−/−^ mice, whereas much less ROS signals fluorescence was observed in the atherosclerotic lesions in NR1-treated ApoE^−/−^ mice. These data indicate that NR1 treatment has a significant effect on alleviating oxidative stress in the atherosclerotic mouse model.

### Effects of NR1 on the serum lipid level

To evaluate the effect of R1 on the lipid metabolism, the serum concentrations of CHO, TG, LDL and HDL were measured. As shown in **Table 1**, significant increase in the levels of serum CHO (*p*<0.01), TG (*p*<0.01) and LDL (*p*<0.01) was observed in vehicle-treated ApoE^−/−^ mice compared to that from the vehicle-treated WT controls. The level of HDL exhibited a significant reduction in vehicle-treated ApoE^−/−^ mice compared to that from the vehicle-treated WT controls (*p*<0.01). Compared to that from the vehicle-treated ApoE^−/−^ mice, the levels of CHO (p<0.01) and TG (*p*<0.01) were significantly decreased in NR1-treated ApoE^−/−^ mice although the level of LDL was not significantly affected by NR1 treatment (*p*>0.05). Meanwhile, the level of HDL in ApoE^−/−^ mice was significantly increased by NR1 treatment compared to that from the vehicle-treated ApoE^−/−^ mice (*p*<0.05). Moreover, NR1 treatment led to a significant reduction in the level of ox-LDL in ApoE^−/−^ mice (*p*<0.01), which was remarkably increased in the vehicle-treated ApoE^−/−^ mice compared to that from the vehicle-treated WT controls (*p*<0.01). These results indicate that NR1 treatment exert significant effect on modulating lipid profile in the atherosclerotic mouse model.

**Table 1:** Levels of the serum lipids.

Lipid	WT	ApoE^−/−^	ApoE^−/−^NR1
CHO (mmol/L)	6.46±0.55	15.87±0.39#	12.96±0.78**
TG(mmol/L)	0.84±0.05	2.82±0.08#	2.39±0.06**
LDL (mmol/L)	4.14±0.24	10.22±0.36#	9.90±0.38
HDL(mmol/L)	0.84±0.02	0.41±0.02#	0.51±0.04*
ox-LDL (ug/L)	31.10±0.96	73.07±1.29#	50.74±0.69**

# ApoE^−/−^ vs. WT, *p*<0.01.

* ApoE^−/−^NR1 vs. ApoE^−/−^, *p*<0.05.

** ApoE^−/−^NR1 vs. ApoE^−/−^, *p*<0.01. Statistical analysis was first performed by one-way ANOVA to test the difference among all three groups, which was followed by Student's *t* test to compare the difference between ApoE^−/−^ vs. WT and ApoE^−/−^NR1 vs. ApoE^−/−^, respectively.

### The effect of NR1 on the production of inflammatory cytokines

Additionally, the levels of serum IL-2, IL-6, TNF-α and γ-IFN were measured to assess the effect of NR1 on inflammatory cytokine production in ApoE^−/−^ mice. As shown in **Table 2**, compared to that from the vehicle-treated WT mice, the levels of IL-2, IL-6, TNF-α and γ-IFN were significantly increased in vehicle-treated ApoE^−/−^ mice (*p*<0.01). In distinct contrast, NR1 treatment significantly reduced the levels of these pro-inflammatory cytokines in ApoE^−/−^ mice (*p*<0.01), suggesting that NR1 treatment significantly alleviated the inflammatory response in the atherosclerotic mouse model.

**Table 2:** Levels of the pro-inflammatory cytokines.

Cytokines	WT	ApoE^−/−^	ApoE^−/−^NR1
IL-2 (pg/mL)	14.86±0.68	41.74±0.68#	30.99±0.58*
IL-6 (ng/L)	70.51±3.11	174.40±1.91#	136.74±5.66*
TNFα (ng/L)	395.33±11.85	715.15±8.40#	566.26±30.90*
γ-IFN (ng/L)	1071.59±37.12	2329.33±62.88#	1643.14±39.78*

# ApoE^−/−^ vs. WT, *p*<0.01;

* ApoE^−/−^NR1 vs. ApoE^−/−^, *p*<0.01. Statistical analysis was first performed by one-way ANOVA to test the difference among all three groups, which was followed by Student's *t* test to compare the difference between ApoE^−/−^ vs. WT and ApoE^−/−^NR1 vs. ApoE^−/−^, respectively.

### Effects of NR1 on the expression of miRNAs implicated in atherosclerotic lesions

To further address the mechanisms that may contribute to the interventional effects of NR1 on atherosclerosis, the expression of an array of miRNA known to play important roles in various aspects of atherosclerosis was analyzed by real-time PCR. Among all the miRNAs examined, significantly upregulated expression of miR-21, miR-26a, and downregulated expression of miR-20 was observed in aortas in NR1-treated ApoE^−/−^ mice compared to that from the vehicle-treated ApoE^−/−^ mice (**Table 3**), suggesting that NR1 may exert the anti-atherosclerotic effects in part through modulating the expression of regulatory miRNAs in atherosclerosis.

**Table 3:** Expression level of the miRNAs.

miRNA name	WT	ApoE^−/−^	ApoE^−/−^NR1
mmu-miR-20a-5p	1.00±0.08	0.53±0.05#	1.22±0.16*
mmu-miR-21a-5p	1.00±0.10	4.07±0.49#	0.96±0.12*
mmu-mir-26a-5p	1.00±0.12	2.38±0.24#	1.37±0.30*
mmu-miR-92a-3p	1.00±0.09	0.75±0.03#	0.95±0.12
mmu-miR-126a-3p	1.00±0.12	5.64±0.20#	1.82±0.36*
mmu-miR-132-3p	1.00±0.14	3.54±0.09#	3.03±0.34
mmu-miR-146a-5p	1.00±0.27	2.81±0.45#	1.66±0.63
mmu-miR-155-5p	1.00±0.04	2.67±0.40#	1.67±0.30

# ApoE^−/−^ vs WT, *p*<0.05;

* ApoE^−/−^NR1 vs ApoE^−/−^, *p*<0.05. Statistical analysis was first performed by one-way ANOVA to test the difference among all three groups, which was followed by Student's *t* test to compare the difference between ApoE^−/−^ vs. WT and ApoE^−/−^NR1 vs. ApoE^−/−^, respectively.

## Discussion

Although effective pharmacological agents are available in lowering the atherogenic lipid protein and have been benefiting the clinical management of atherosclerosis, cardiovascular disease and stroke remain the most common causes of mortality worldwide. Therefore new therapies equipped with the capacity to target complex pathological changes occurring at the site of the vessel wall or raise anti-atherogenic HDL, are in need to further enhance the clinical outcome of atherosclerotic patients. In our current study, we reported that NR1 treatment attenuated atherosclerotic lesions in ApoE^−/−^ mice. The anti-atherosclerotic effect of NR1 could not only be attributed to lower levels of atherogenic lipid protein and higher level of HDL, it could also result from decreased level of oxidative stress and inflammation. Moreover, NR1 treatment led to altered vascular expression of several miRNAs that are implicated in the pathogenesis of atherosclerosis.

NR1 treatment resulted in not only decreased level of CHO and TG, but also increased amount of HDL in the serum. These data suggest that the anti-atherosclerotic effects of NR1 could in part result from its action on modulating the lipid metabolism in ApoE^−/−^ mouse model. Panax notoginseng and its saponins have been previously shown to be able to regulate the lipid metabolism [21]. Therefore this effect of NR1 on lipid proteins suggests that NR1 could at least be one of the major active constituents that confer the effects of PNS on lipid regulation in the context of atherosclerosis.

In addition, our results indicate that NR1 treatment led to decreased levels of MDH increased level of SOD and GSH in the blood. It is worth noting that the level of ox-LDL but not LDL was significantly decreased by NR1 treatment, further supporting its effect on alleviating oxidative stress. Meanwhile, Significantly decreased amount of inflammatory cytokines such as IL-2, IL6, TNF-α and γ-IFN was observed after NR1 treatment. At the site of atherosclerotic lesions, much less F4/80 positive macrophages and α-SMA positive VSMC was detected after NR1 treatment (**Figure S2**). These results collectively support the notion that NR1 mitigates the development of atherosclerotic pathologies in part through attenuating inflammation, oxidative stress and atherosclerotic fibrosis. The anti-oxidative and anti-inflammatory activities of NR1 have been demonstrated to be responsible for its neuroprotective and cardioprotective effects. Studies in ischemic brain have shown that as a phytoestrogen, NR1 treatment suppresses oxidative stress through inhibiting NADPH oxidase activity via estrogen receptor (ER) dependent activation of Akt2/Nrf2 pathway [22], [23]. Moreover, NR1 protects the heart from septic shock in part by activating ERα and PI3K/Akt signaling, resulting in the inhibition of NF-κB-mediated transcription and attenuation of the pro-inflammatory response in the myocardium [16]. It is also worth noting that ER signaling is implicated in the pathogenesis of atherosclerosis. Estrogen binds ER in VSMC and has been postulated as an anti-atherogenic agent through inhibiting VSMC proliferation [24]. Decreased levels of ERs, in particular, ERα have been implied to be associated with the progression of atherosclerosis. Moreover, enhanced methylation of ERα has been noted in coronary atherosclerotic plagues and this methylation-associated gene inactivation of ERα in vascular tissue may mechanistically contribute to atherogenesis [25]. Therefore, it is worth pursuing in our future studies to test the hypothesis that as a phytoestrogen, NR1 may exert the anti-atherosclerotic effects in part through modulating ER signaling during atherosclerosis.

miRNAs are important regulators implicated in each step of the cascade that leads to atherosclerotic lesion formation and progression [10]. Our results revealed altered expression of an array of miRNAs in the atherosclerotic vessel wall that regulates inflammation, oxidative stress, angiogenesis and fibrosis (**Table 3**). Once infiltrate into the vessel wall, monocytes differentiate into macrophages and this process is partly promoted or inhibited by miRNAs. miR-155 is pro-differentiative [26] and miR-20a has been revealed to be anti-differentiative [27]. In relation to atherosclerosis, miR-155 also inhibits TGF-β dependent differentiation and promotes the proliferation of VSMCs [28]. miR-146a could be induced by ox-LDL and stimulates the production of pro-inflammatory cytokines [29]. miR-21a increases ROS formation partly through repressing SOD2, an protein critical for mitochondrial oxidative defense [30]. miR-21a is also able to stimulate VSMC proliferation in vitro and neointimal formation in vivo by downregulating PTEN and Bcl-2 [31]. miR-26a induces the proliferation and migration while inhibits the differentiation and apoptosis of VSMCs by negatively regulating TGF-β signaling, thereby is implicated in VSMC-mediated fibrotic events that promote atherosclerotic progression [32]. Aberrant angiogenesis is a harmful event in the pathogenesis of atherosclerosis. Proangiogenic miR-126a [33] and miR-132 [34] and anti-angiogenic miR-92a [35] could be implicated in plaque angiogenesis, which contributes to destabilization and rupture of atherosclerotic lesions and may also lead to increased accumulation of inflammatory cells. Significantly increased aortic expression of miR-146a, miR-26a, miR-21a, miR-155, miR-126a and miR-132, decreased expression of miR-20a and miR-92a was observed in vehicle-treated ApoE^−/−^ mice. It is worth noting that NR1 treatment significantly decreased the expression of miR-21a, miR-26a, miR-126a and increased the expression of miR-20a in the aortas, suggesting that the anti-atherosclerotic effects of NR1 could be mediated in part through modulating the expression of miRNAs implicated in monocyte differentiation, oxidative stress, fibrosis as well as angiogenesis. These results validate the anti-atherosclerotic effects of NR1 at the molecular level, suggesting that the anti-atherosclerotic effect of NR1 treatment could be associated with the changes in miRNA expression. Further studies are required to address whether the altered expression of the related miRNAs causally contributes to the interventional effects of NR1 on atherosclerotic lesion development.

## Conclusions

In summary, our results demonstrated for the first time the effect of NR1 in alleviating the atherosclerotic lesions in ApoE^−/−^ mouse model. The experimental evidence was also provided to support the notion that such an anti-atherosclerotic action of NR1 may be attributed to its activities in attenuating lipid abnormality, inflammation, oxidative stress and atherosclerotic fibrosis. Furthermore, NR1 treatment exerts significant effects on modulating the expression of miRNAs that play important roles in the pathogenesis of atherosclerosis. Collectively, our data demonstrated that NR1 is able to achieve the anti-atherosclerotic effects by targeting multiple mechanisms, which offers the possibility of developing NR1-based therapeutic strategy in treating atherosclerosis.

## Supporting Information

Figure S1 NR1 treatment alleviated the atherosclerotic lesion formation in the thoracic aorta in ApoE^−/−^ mice. Serial paraffin-embedded sections of the thoracic aorta from vehicle-treated WT control (**a**), vehicle-treated ApoE^−/−^ mice (**b**) and NR1-treated ApoE^−/−^ mice (**c**) were stained with H&E and observed under light microscopy. The size of atherosclerotic lesions in vehicle-treated ApoE^−/−^ mice and NR1-treated ApoE^−/−^ was measured and quantified (**d**). * ApoE^−/−^NR1 vs. ApoE^−/−^, *p*<0.05. Scale bar = 200 µm.
(TIF)

Figure S2 NR1 alleviated VSMC and macrophage presence in the atherosclerotic lesions in the ApoE^−/−^ mice. **A**. Sections of aortic roots from the vehicle-treated ApoE^−/−^ mice (**a**) and NR1-treated ApoE^−/−^ mice (**b**) were examined for the expression of α-smooth muscle actin in the atherosclerotic lesions. **B**. Sections of aortic roots from the vehicle-treated ApoE^−/−^ mice (**a**) and NR1-treated ApoE^−/−^ mice (**b**) were examined for the expression of F4/80 in the atherosclerotic lesions. Scale bar = 100 µm.
(TIF)

Table S1 Primers sequences for miRNA expression analyses.
(DOCX)
