# Supplementary figures and images for: Notoginsenoside R1 Attenuates Atherosclerotic Lesions in ApoE Deficient Mouse Model
Source: PLoS One. 2014 Jun 16;9(6):e99849. doi: 10.1371/journal.pone.0099849 (PMC4059705; doi:10.1371/journal.pone.0099849)

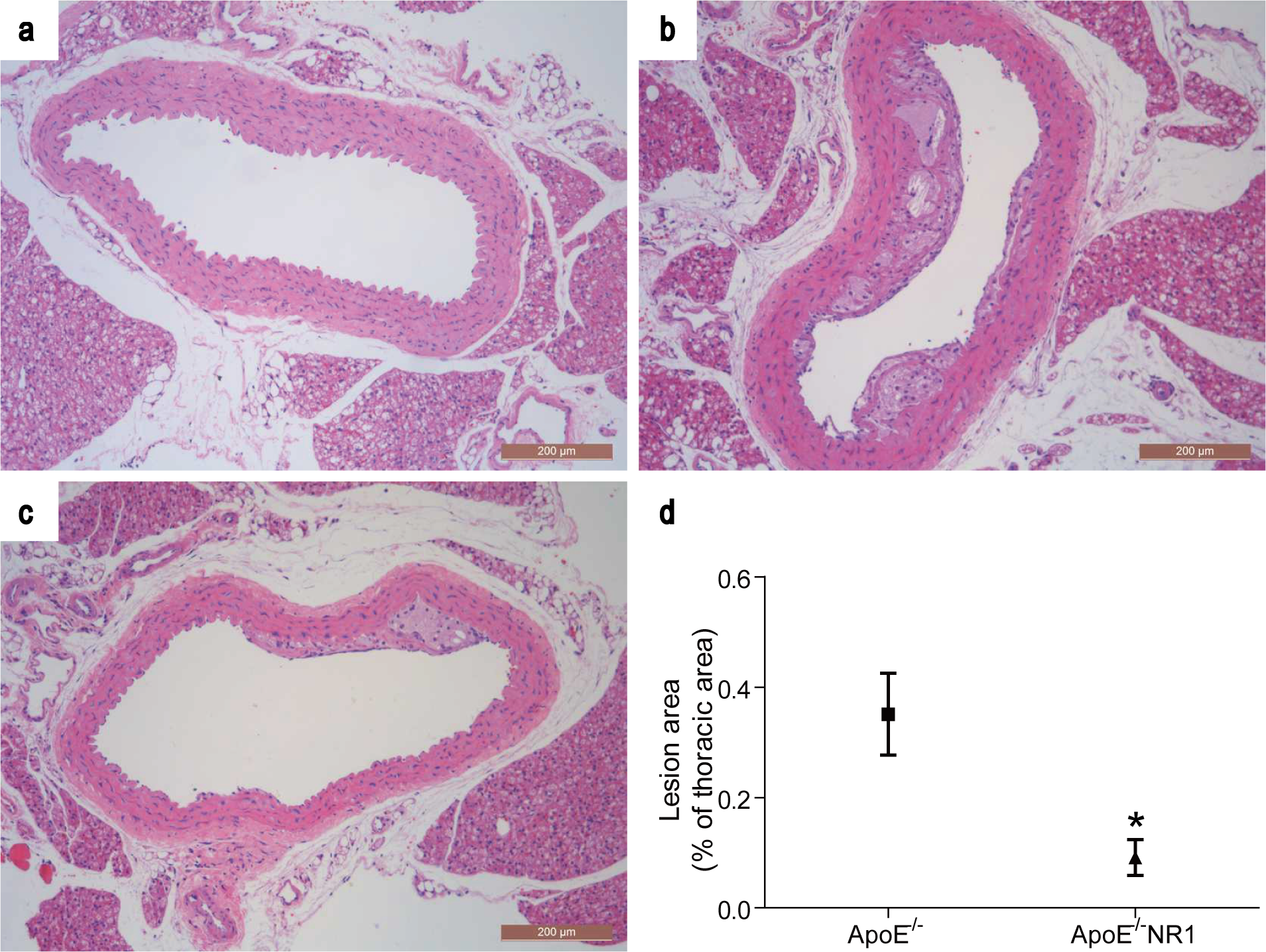

Supplement: Figure S1 — NR1 treatment alleviated the atherosclerotic lesion formation in the thoracic aorta in ApoE−/− mice. Serial paraffin-embedded sections of the thoracic aorta from vehicle-treated WT control (a), vehicle-treated ApoE−/− mice (b) and NR1-treated ApoE−/− mice (c) were stained with H&E and observed under light microscopy. The size of atherosclerotic lesions in vehicle-treated ApoE−/− mice and NR1-treated ApoE−/− was measured and quantified (d). * ApoE−/−NR1 vs. ApoE−/−, p<0.05. Scale bar = 200 µm. (TIF) [file pone.0099849.s001.tif]

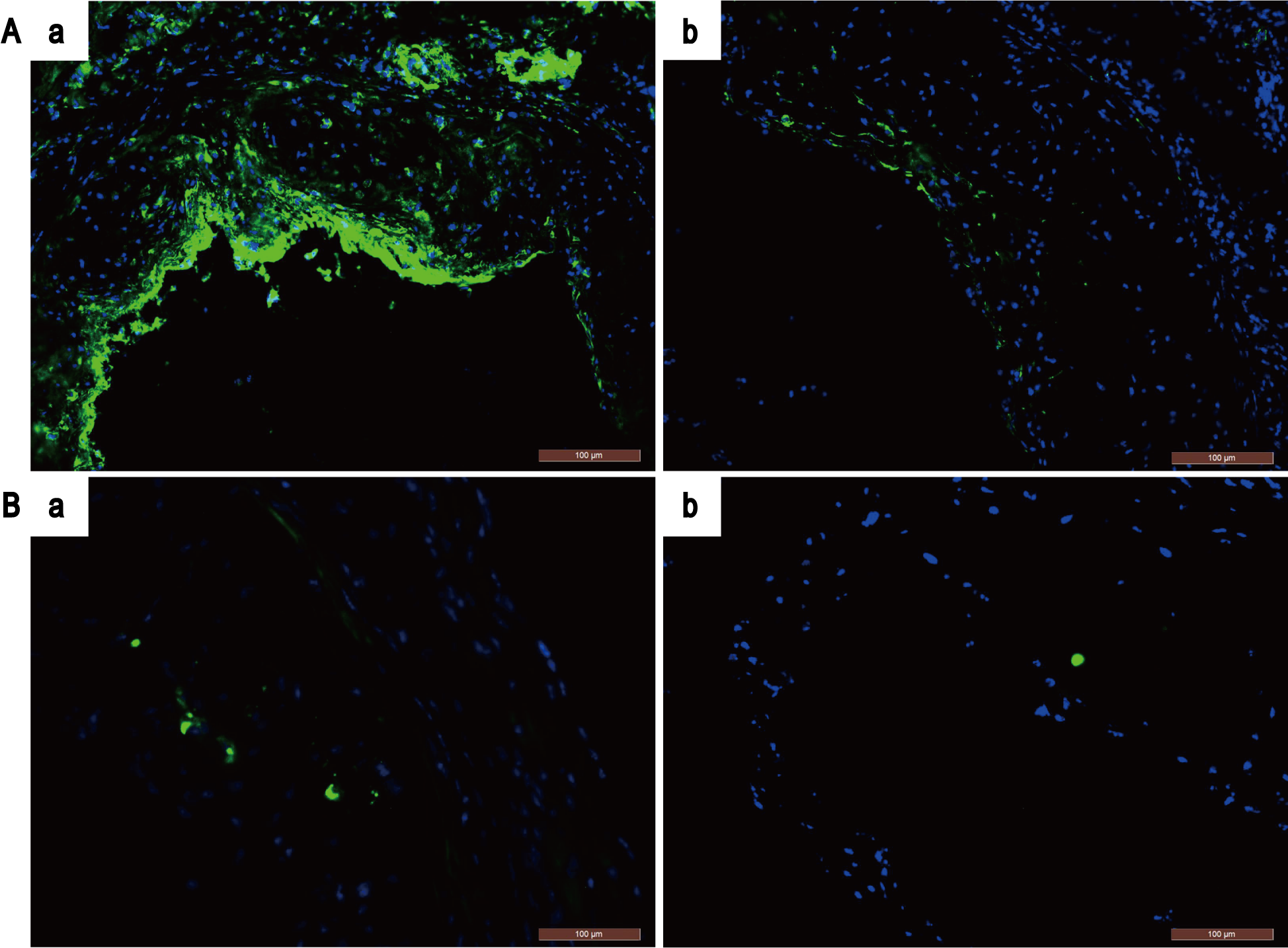

Supplement: Figure S2 — NR1 alleviated VSMC and macrophage presence in the atherosclerotic lesions in the ApoE−/− mice. A. Sections of aortic roots from the vehicle-treated ApoE−/− mice (a) and NR1-treated ApoE−/− mice (b) were examined for the expression of α-smooth muscle actin in the atherosclerotic lesions. B. Sections of aortic roots from the vehicle-treated ApoE−/− mice (a) and NR1-treated ApoE−/− mice (b) were examined for the expression of F4/80 in the atherosclerotic lesions. Scale bar = 100 µm. (TIF) [file pone.0099849.s002.tif]
