# Supplementary material for: Notoginsenoside R1 Attenuates Atherosclerotic Lesions in ApoE Deficient Mouse Model
Source: PLoS One. 2014 Jun 16;9(6):e99849. doi: 10.1371/journal.pone.0099849 (PMC4059705; doi:10.1371/journal.pone.0099849)
Supplement: Table S1 — Primers sequences for miRNA expression analyses. (DOCX) [file pone.0099849.s003.docx]

**Table S1. Primers sequences for miRNA expression analyses**

| miRNAs name | Primer sequences from 5’ to 3’ |
| --- | --- |
| mmu-miR-20a-5p | CGTAAAGTGCTTATAGTGCAGGTAG |
| mmu-miR-21a-5p | CGTAGCTTATCAGACTGATGTTGA |
| mmu-miR-26a-5p | TTCAAGTAATCCAGGATAGGCT |
| mmu-miR-92a-3p | TGCACTTGTCCCGGC |
| mmu-miR-126a-3p | CGTACCGTGAGTAATAATGCG |
| mmu-miR-132-3p | TAACAGTCTACAGCCATGGTCG |
| mmu-miR-146a-5p | TGAGAACTGAATTCCATGGG |
| mmu-miR-155-5p | TTAATGCTAATTGTGATAGGGGT |
| 5S | GATCTCGTCTGATCTCGGAAGC |
